# Understanding COVID-19 vaccine hesitancy in the Hispanic adult population of South Carolina: a complex mixed-method design evaluation study

**DOI:** 10.1186/s12889-023-16771-9

**Published:** 2023-11-28

**Authors:** Maria Mercedes Rossi, Michelle A. Parisi, Kathleen B. Cartmell, Danielle McFall

**Affiliations:** 1 Department of Food, Nutrition, and Packaging Sciences, C228 Pool Agricultural Center, Clemson University, Clemson, SC 29631 USA; 2 Department Nutritional Sciences, University of Georgia, 206 Hoke Smith Annex, Athens, GA 30602 USA; 3 Department of Public Health Sciences, Clemson University, 534 Edwards Hall, Clemson, SC 29634 USA; 4 Rural Health and Nutrition Program Team, Clemson University, 120 Lehotsky Hall, Clemson, SC 29634 USA

**Keywords:** Vaccine hesitancy, COVID-19, Attitudes, Barriers, Participants’ perceptions, Focus groups, Mixed method

## Abstract

**Background:**

In August 2021, only 47.6% of all eligible residents in South Carolina (SC) had received at least one dose of the COVID-19 vaccine, with only 41% having completed their vaccination series. Additionally, only 27% of all Hispanics in SC had completed their vaccination series compared to 34.1% of non-Hispanics. Vaccine hesitancy is a complex phenomenon that is context and vaccine-specific. Focusing on unvaccinated Hispanics living in rural areas of SC, this study aimed to identify barriers to vaccination and provide an educational intervention designed to address vaccine hesitancy.

**Methods:**

A complex mixed-methods evaluation design was used to conduct this study. First, in-person vaccine educational sessions were implemented, along with a pre-post-test survey, to assess changes in knowledge, attitudes, motivations, barriers, and intentions to receive COVID-19 vaccination. Second, in-person follow-up focus groups were held with the same participants to gather in-depth insight about participants’ knowledge and attitudes about the COVID-19 vaccination. Third, an online follow-up survey was conducted to assess the effect of the training and discussion session on COVID-19 vaccination. Study outcomes were assessed among the 17 individuals who participated in the educational sessions and focus group discussions.

**Results:**

Findings revealed that for unvaccinated Hispanics living in South Carolina; vaccine hesitancy was primarily driven by: 1) misinformation and information coming from unverified sources and 2) negative perceptions of the safety and effectiveness of the COVID-19 vaccines. Specifically, participants were fearful that the vaccine development was rushed and that the vaccines might contain questionable ingredients that could cause strong side effects or even death. Participants were also concerned that vaccination might cause them to get sick and be hospitalized, which would have financial implications since they could not afford healthcare or take time off work.

**Conclusions:**

Program implementation and mass communication campaigns should focus on COVID-19 vaccine safety and effectiveness, including side effects, what to expect after being vaccinated, and how to look for information from reputable sources. The educational session implemented proved to be effective and helped reduce vaccine hesitancy since most participants (80%) self-reported receiving a COVID-19 vaccine after program participation.

**Supplementary Information:**

The online version contains supplementary material available at 10.1186/s12889-023-16771-9.

## Background

Herd immunity is achieved when most of a population becomes immune to a disease either through natural infection or vaccination, protecting against it [1]. The main goal is to protect people from getting COVID-19 and reduce its severity [1]. Vaccine hesitancy is defined as delaying or refusing a vaccine despite its availability [2], and it is more prevalent with new vaccines like the COVID-19 vaccine. It is a complex social and behavioral phenomenon that is vaccine and context-specific [2].

By the end of April 2021, when all US adults were eligible to receive a COVID-19 vaccine, vaccination coverage was highest among White (59.0%) adults and was lower among Hispanics (47.3%) and Blacks (46.3%) [3]. According to the SC State of Profile Report, in August 2021, by the time this research project was conducted, 47.6% of all eligible residents in SC had received at least one dose of the COVID-19 vaccine, while 41% had completed their vaccination series [4]. In contrast, 27% of all Hispanics in SC had completed their vaccination series compared to 34.1% of non-Hispanics [5]. Furthermore, 27% of Blacks had completed their vaccination series, while 32.6% of White South Carolineans had completed it [6]. The combination of observed COVID-19 vaccine hesitancy and racial disparities in vaccination rates has prompted many scholars to research and develop interventions focusing on minorities. However, education interventions targeted to Hispanics living in rural areas to address vaccine hesitancy and researchers that aimed to identify barriers to vaccination have been limited [7].

Hispanics in rural areas often face barriers to COVID-19 vaccination, linked to unreliable sources of information, social media misinformation, and mistrust [8, 9]. A study showed Hispanics had wrong or harmful information about the COVID-19 vaccine shared directly with them through Facebook [10]. Factors contributing to their hesitancy include concerns about vaccine safety, mistrust, and the need for recommendations from same-race medical professionals. However, conflicting vaccine information on platforms, news, and unclear scientific backing from leaders can pose problems [11]. Compounding the problem, many people, including doctors and nurses, use social media platforms to share their personal opinion, which is not necessarily based on scientific information.

Hispanics perceived themselves as healthy but lacked resources on the COVID-19 vaccine in Spanish and were concerned about possible deportation [12]. Undocumented immigrants were also worried about possible deportation and DNA alteration; they believed vaccines might also lead to sterilization. They also worried that side effects from the vaccine might affect their ability to work and expressed concerns about vaccine cost [12]. Kricorian and Turner [8] assessed the COVID-19 vaccine acceptance and beliefs among Black and Hispanic Americans across the US. Most Blacks (55.1%) and Hispanics (49.3%) were significantly less likely than Whites (38.7%) to believe that the vaccine would be effective. Both Hispanics and Blacks were concerned that the COVID-19 vaccine might be unsafe and not worth the risk. For them, it was vital that a medical professional of the same race/ethnicity recommend the vaccine before following through with vaccination [8].

Schilling et al. [9] examined parental intentions to vaccinate their children against COVID-19. Hispanics had the highest vaccination intent (64%) for their children, followed by 16% White Non-Hispanics, and 14% Non-Hispanic Blacks. The results of the study showed that the changing COVID-19 disease and vaccine recommendations and misinformation affected parental vaccine decisions about vaccinated their children. Parental hesitancy is rooted in the perception of the vaccine efficacy, safety, and unspecified risks; and the perception of lower disease severity among children [9]. McFadden et al. [12] found that Hispanics lacked resources about the COVID-19 vaccine in Spanish. The literature highligts similar perceptions about COVID-19 vaccines among immigrant and non-immigrants, and English Proficient Hispanics. Both documented and undocumented immigrants feared deportation when seeking vaccination. Regardless of their immigration status, their concerns also encompassed vaccine-related DNA changes and sterilization fears, potential work interference due to side effects and worries about the vaccine cost. Moreover, there was a prevalent worry that side effects from the vaccine might affect work capabilities and expressed concerns about financial implications of vaccination [12]. Interestingly, some research outcomes contradicted these concerns, indicating that adults from immigrant families exhibited a stronger vaccination intent (75%) in comparison to non-immigrant adults (68%). García et al. [13] conducted a qualitative study with Hispanic families. The study results revealed that vaccine hesitancy was associated with a lack of information, side effects, and mistrust of doctors [13]. These results about correlates of vaccine hesitancy are consistent with other studies published on vaccine hesitancy in Hispanics [9, 11, 13].

To further contribute to understanding about vaccine hesitancy in rural Hispanics, this study focuses specifically on unvaccinated rural Hispanics in SC, aiming to address vaccine hesitancy through an educational intervention and understand reasons for vaccine hesitancy through focus group discussions..

## Methods

### Study design

A complex mixed methods evaluation design was utilized to study Hispanic population vaccine hesitancy in rural counties of South Carolina. This type of design intentionally incorporates quantitative and qualitative data analysis to produce robust results [14]. The study was implemented in Spanish in four different locations. First, the lead researcher moderated the in-person COVID-19 vaccine educational sessions, then a pre-and post-test survey were implemented to assess changes in knowledge, attitudes, motivations, barriers, and intentions to receive a COVID-19 vaccine. Immediately following the post-test, focus groups were held with the same participants to gather more in-depth information about beliefs and attitudes toward COVID-19 vaccination. Between 6 and 8 months after the education and focus group session, participants were contacted and asked to complete an online follow-up survey to assess the effect of the education and focus group session on completion of COVID-19 vaccination. Study outcomes were assessed among the 17 previously unvaccinated individuals in the focus group and educational session. Inclusion criteria included age (adults 18 years or older), vaccination status (unvaccinated), primary language spoken at home (Spanish), and county of residence (living or working in identified priority counties). Participants from Greenville, Oconee, Spartanburg, Lexington-Saluda Counties of South Carolina were included in the study. The Clemson Univeristy (CU) Institute Review Board (IRB) approved the research study (IRB2021-0621).

### Procedures

We completed the pre-post test and focus groups discussions from August to October 2021, while the online follow-up survey was carried out from March to April 2022.

### Sample

To recruit participants for the study, we utilized a homogeneity sampling technique. To assist recruitment, we contacted different people and non-profit organizations within the Hispanic community via email, phone calls, or Zoom meetings. Twenty-three Hispanic adults participated in the study. However, six reported being recently vaccinated for COVID-19 during the pre-survey and were excluded from the data analysis. Four focus groups were conducted, which included 17 total unvaccinated participants, with 2, 4, 5, and 6 participants, respectively, per group.

Enrolled participants were given a $10 pre-paid credit card to offset the cost of gas during the pandemic. A meal was provided for focus groups scheduled during regular mealtimes, and childcare was provided to further address potential participation barriers. The educational session and focus group discussions were completed between.

In person COVID-19 vaccine educational sessions were implemented in Spanish by the lead researcher in Oconee County, Greenville County, Lexington County, and Spartanburg County with a pre-and post-test survey to assess changes in knowledge, attitudes, motivations, barriers, and intentions to receive a COVID-19 vaccine. Upon arrival, participants were provided with information about the study and asked to sign IRB-approved informed consent information. Informed consent was in Spanish and read aloud to participants when needed.

The pre-test was written in Spanish and distributed to participants, and it took between 10–15 min for completion. Items on the pre-test included: 1) likelihood of getting a COVID-19 vaccine, 2) If getting a COVID-19 vaccine, when you plan to get it, 3) participants’ agreement with statements regarding the safety and effectiveness of the COVID-19 vaccines, 4) reasons for getting a COVID-19 vaccine, 5) reasons for not getting a COVID-19 vaccine, 6) knowledge questions about the COVID-19 vaccine and 7) participants demographic information. Items were read aloud to participants as needed. Table 1 shows the pre-post-test questions and response options.

**Table 1:** Clemson cooperative extension service questionnaire on COVID-19 vaccine-pre-test

Item	Responses options
**How likely do you think you are to get a COVID-19 vaccine?**	I have already gotten a COVID-19 vaccine Very Likely Likely Unsure Unlikely Very Unlikely
**If you think you may get a vaccine, when do you plan to get vaccinated?**	I have already gotten a COVID-19 vaccine Within the next month Within the next 1 to 3 months Within the next 3 to 6 months Within the next 6 to 12 months I plan to wait a year or longer to get the vaccine I do not plan to get a COVID-19 vaccine
**For the statements below, please circle the response that best describes how you feel about each statement:** - I think the COVID-19 vaccines are safe - I think the COVID-19 vaccines are effective - I feel confident in how the COVID-19 vaccines were developed - I feel confident in the way the government is ensuring the safety of the COVID-19 vaccines	Agree Not sure Disagree
**If you were to get the COVID-19 vaccine, why would you get it? *(Check all that apply)***	I do not plan to get the vaccine I want to protect a family member or close friend that is high-risk for severe disease I want to protect myself from getting COVID-19 I am concerned about possible virus exposures at work or school I am concerned about possible virus exposures in my community I want to do my part to help control the pandemic I want to serve as an example to encourage others to take the vaccine I want to travel and do the things I enjoyed before the pandemic Only if my employer requires COVID-19 vaccination Other: Please specify
**If you are not planning to get the vaccine or if you are still undecided, what are the reasons for not getting a COVID-19 vaccine? *(Check all that apply)***	I do not think that I am at risk for serious disease I do not believe I need the vaccine because I already had COVID-19 infection I am not eligible to get the COVID-19 vaccine based on my medical conditions (immunocompromised, pregnant, or as advised by a doctor) I have religious, or cultural beliefs against the COVID-19 vaccine I do not think the vaccine is safe If you think the vaccine is not safe, why? I do not think the vaccine will protect me from getting sick with COVID-19 I do not have time or can not miss work to take the vaccine I can not afford to pay for the vaccine I do not have transportation to get the vaccine I have a fear of needles I have concerns about attending clinics where law enforcement and military representatives are present I don’t trust the healthcare providers or government organizations who are offering the vaccine I am waiting to see how other people are affected by the vaccine Other: Please specify:
**If you are interested in learning more about the COVID-19 vaccine or in getting a COVID-19 vaccine, what information or resources would be helpful?**	Open ended question
***Please Respond to the questions below by choosing true or false for each statement*** - People under the age of 65 don’t need to get the vaccine - If I’ve already had COVID-19, I don’t need to get the vaccine - The COVID-19 vaccine cannot alter my DNA because it does not interact with DNA in the body - There is no evidence that the vaccine will decrease fertility (ability to have children) - After receiving the COVID-19 vaccine, I could test positive for COVID-19 because the vaccine contains the live COVID-19 virus - The COVID-19 vaccine can make me magnetic because it contains metal - Getting the vaccine can cause me to get COVID-19 disease - You must have insurance to get the vaccine - The vaccine is free - You do not have to show citizenship documentation to get the vaccine	True or False

### Educational session with pre-post survey

The one-time brief education session, which was delivered to each of the four groups participants, took approximately 20 min. It addressed the eleven most common myths regarding the COVID-19 vaccines. Each participant received a postcard with myths, read them aloud, and shared their opinion about them. The researcher prompted discussion about the myth and concluded by providing the fact behind that myth. Participants also watched a video in Spanish prepared by the CDC about vaccinating with confidence. The video concluded by encouraging the viewer to look for more information about the COVID-19 vaccine from trusted sources such as the CDC in Spanish. Additionally, the lead researcher addressed how the mRNA vaccine works and the possible side effects of vaccination using flyers developed by the CDC in Spanish. At the conclusion of the education delivery, a post-test was distributed. The post-test was an exact copy of the pre-test. All participants completed the post-test survey. Participants were asked to stay for the focus group portion of the study.

### Focus group sessions

A Focus group discussion is a qualitative technique designed to gain an in-depth understanding of a specific topic of interest [15]. Despite participants answering the facilitator’s questions individually, they are prompted to speak and interact with one another [15]. In-person, 60-min focus group discussion was used to gather in-depth perceptions of the COVID-19 vaccine and participants’ attitudes toward vaccination. After completing the focus group discussions, participants were given information about local options to receive a COVID-19 vaccine. Contact information to make appointments and addresses of vaccination sites were provided. Participants were then invited to stay for a meal and were given a pre-paid gift card to account for gas used to and from the session and to travel to get vaccinated if they chose to do so.

### Online follow-up survey

Six to 8 months after the education and focus group sessions, an online self-reported follow-up survey was conducted using Qualtrics XM to assess the long-term effect of the education and focus group session on COVID-19 vaccination status. Study outcomes were assessed for the 17 previously unvaccinated individuals who participated in the the study. Three of the 17 participants were lost to follow-up because of apparent changes in contact information since the time of education and focus group participation. Therefore, 14 individuals were contacted and asked to complete the online survey. Participants were provided with the survey link via email or phone text message. Of the initial 17 participants who completed the education session and focus group discussions, 10 participants completed a self-reported online follow-up survey.

### Data analysis

Pre-post-test surveys were analyzed using Microsoft Excel for Mac (Version 16.74). Frequencies and percentages were calculated with the pivot table function with pre-post-test results assessed for each each statement. Focus group responses were translated to English verbatim and then edited for readability by filling in missing words and correcting spelling and typographical errors. Following the methodology described by Saldaña, a codebook was created, and final codes were agreed upon for data analysis [16]. Two researchers MMR and KBC, worked to create a codebook and agreed upon final codes for analyzing the data. They also developed each code definitions. As a second step, the researchers discussed and confirm codes. Differences in coding were examined until a consensus was reached [16]. As a final step, codes were reviewed to identify themes and sub-themes across the focus group discussions. Saturation was reached, as the researchers got to the point where no new information emerged during the coding [16]. The online follow-up survey was also analyzed using Microsoft Excel for Mac (Version 16.74). Frequencies and percentages were calculated with the pivot table function to report participants’ vaccination status (no vaccination, first-dose vaccine completion, and vaccine series completion).

## Results

All participants were from Oconee County (35%), Lexington County (29%), Spartanburg (24%), and Greenville (12%), and most were female (76%). Participants were 18–27 (24%) years old, 28–37 (24%), 38–47 (24%), 48–57 (6%), 58–67 (6%), and more than 68 years old, respectively. Table 2 summarizes participants’ demographic characteristics.

**Table 2:** Participants’ demographic characteristics (*n* = 17)

Characteristics	f	Percentage
County		
Oconee	6	35%
Lexington	5	29%
Spartanburg	4	24%
Greenville	2	12%
Years of age (ranges)		
18–27 years old	4	24%
28–37 years old	4	24%
38–47 years old	4	24%
48–57 years old	1	6%
58–67 years old	1	6%
More than 68 years old	1	6%
Not reported	2	12%
Gender		
Female	13	76%
Male	4	24%

### Results. Pre-post-test conducted before and after educational session

Regarding their intentions of getting a COVID-19 vaccine, 12% of participants reported being very likely to get the vaccine during pre-test. During post-test, 29% were very likely, reflecting a 17% increase in the likelihood of vaccinating. Also, 29% reported being very likely to get a COVID-19 vaccine during pre-test. At post-test, 59% said being likely, reflecting a 30% increase in the likelihood of vaccination. Only a few participants (29%) were unsure about being vaccinated during the pre-test. Only 12% reported being unsure at post-test, showing a decrease of 17% in their likelihood of vaccination. Table 3 summarizes participants’ likelihood of getting vaccinated against the COVID-19 disease.

**Table 3:** Descriptive statistics: participants’ likelihood of getting a COVID-19 vaccine

Response options	Pre-Test		Post-Test		Difference
	f	%	f	%	
Very Likely	2	12%	5	29%	+17%
Likely	5	29%	10	59%	+30%
Unsure	5	29%	2	12%	-17%
Unlikely	4	24%	0	0%	-24%
Very Unlikely	1	6%	0	0%	-6%

Participants were asked about their intent to receive a COVID-19 vaccine and timeframe for completing vaccination. Those who responded that they did not plan to get a COVID-19 vaccine at pre-test (6%) did not change their intentions at post-test (6%). There was also no change for those who said they would get a COVID-19 vaccine within the next 3 months (6%). However, at pre-test, 24% said they would get a vaccine within a month. On the contrary, 35% said they would get it within a month at post-test, showing an 11% increase in their intentions to vaccinate within a month. Table 4 synthesizes participants’ plans to get vaccinated.

**Table 4:** Descriptive statistics: participants’ plans for getting a COVID-19 vaccine

Items	Pre-Survey		Post-Survey		Difference
	f	%	f	%	
I do not plan to get a COVID-19 vaccine	1	6%	1	6%	0%
I plan to wait a year or longer to get the vaccine	4	24%	1	6%	+18%
Within the next month	4	24%	6	35%	+11%
Within the next 1 to 3 months	5	29%	6	35%	+6%
Within the next 3 to 6 months	1	6%	1	6%	0%
Within the next 6 to 12 months	0	0%	1	6%	+6%
Missing Data	2	12%	1	6%	-6%

Percentages might not add up to 100% due to rounding

Regarding the safety of the vaccine, there was no change in how most people (71%) felt about it; they were not sure at pre-and-post-test. Only, 12% thought the vaccines were safe at the pre-test. However, at post-test, 24% thougth they were safe, showing an increase of 12% in those who believed vaccines were safe. Regarding its effectiveness, most participants (71%) felt unsure about the vaccine’s effectiveness at pre-test. In comparison, only 53% felt unsure at post-test, showing a decrease of 17% of those who felt unsure about the vaccine’s effectiveness. Further, 12% agreed about the vaccine’s effectiveness at pre-test. In contrast, 35% agreed after participation, reflecting a 23% increase in participants agreeing about its effectiveness.

Regarding participants’ confidence in how the COVID-19 vaccines were developed, 71% were unsure at pre-test. In contrast, 53% were unsure at post-test, reflecting a decrease of 18% in the number of people that felt unsure. Also, 18% (*n* = 3/17) felt confident about how the vaccines were developed at pre-test. In comparison, 29% (*n* = 5/17) felt confident at post-test, showing an increase of 11% in their confidence about how vaccines were developed. Regarding participants’ confidence in how the government ensures the safety of the COVID-19 vaccines, 18% felt confident at pre-test, but 35% were confident at post-test—indicating an increase of 17% in their confidence level.

Furthermore, 76% said they were unsure at pre-test, but only 53% were unsure at post-test. This indicates a decrease of 23% in the number of participants that felt unsure about how the government ensures the vaccine’s safety. Table 5 synthesizes participants’ level of agreement regarding the safety and effectiveness of the COVID-19 vaccines, confidence in how the vaccines were developed and the confidence on how the government is ensuring the safety of the vaccines.

**Table 5:** Descriptive statistics: level of agreement about COVID-19 vaccines

	Pre-Test			Post-Test			Difference		
	Agree	Not sure	Disagree	Agree	Not sure	Disagree	Agree	Not sure	Disagree
I think the COVID-19 vaccines are safe	12%	71%	18%	24%	71%	0%	+12%	0%	-18%
I think the COVID-19 vaccines are effective	12%	76%	12%	35%	59%	0%	+23%	-17%	-12%
I feel confident in how the COVID-19 vaccines were developed	18%	71%	12%	29%	53%	6%	+11%	-18%	-6%
I feel confident in the way the government is ensuring the safety of the COVID-19 vaccines	18%	76%	6%	35%	53%	6%	+17%	-23%	0%

Participants shared their main reasons for getting vaccinated. At post-test, 59% wanted to protect a family member or close friend at risk (social benefit), 35% wanted to protect themselves (personal benefit), and 29% were concerned about possible virus exposures at school or work and exposure to the community. The reasons for not getting vaccinated were: 29% did not believe the COVID-19 vaccines were safe, and 18% did not think they were at risk for severe disease. Finally, 18% did not believe the vaccine would protect them from getting sick with COVID-19.

Regarding participants’ knowledge (correct answers) about the COVID-19 vaccines, there was an increase in the number of people responding correctly to each statement. The exception were the following statements: I’ve already had COVID-19; I don’t need to get the vaccine, 82% responded correctly at pre-test, but only 71% responded correctly at post-test—showing a decrease of 11% on those who answered correctly to that question. Also, 88% responded correctly to the statement at pre test that you do not have to show citizenship documentation to get the vaccine. However, only 76% answered correctly at post-test, showing a decrease of 12% of those who answered correctly. Table 6 shows differences in knowledge (correct responses) reported by participants before and after attending the educational session.

**Table 6:** Differences in knowledge (correct responses) reported by participants during pre-post-test

Statement	Pre-test		Post Test		Difference
	f	Correct responses	f	Correct responses	
People under the age of 65 don’t need to get the vaccine **(False)**	9	53%	12	71%	+18%
If I’ve already had COVID-19, I don’t need to get the vaccine **(False)**	14	82%	12	71%	-11%
The COVID-19 vaccine cannot alter my DNA because it does not interact with DNA in the body **(True)**	6	35%	6	35%	0%
There is no evidence that the vaccine will decrease fertility (ability to have children) **(True)**	4	24%	7	41%	+17%
After receiving the COVID-19 vaccine, I could test positive for COVID-19 because the vaccine contains the live COVID-19 virus **(False)**	9	53%	10	59%	+6%
The COVID-19 vaccine can make me magnetic because it contains metal **(False)**	14	82%	16	94%	+12%
Getting the vaccine can cause me to get COVID-19 disease **(False)**	4	24%	5	29%	+5%
You must have insurance to get the vaccine **(False)**	15	88%	16	94%	6%
The vaccine is free **(True)**	15	88%	15	88%	0%
You do not have to show citizenship documentation to get the vaccine **(True)**	15	88%	13	76%	-12%

### Focus group discussions results

Seven significant themes and corresponding subthemes were identified and explained with more detail below: COVID-19 vaccine concerns, feelings of fear, knowledge about the COVID-19 vaccine, suggestions to overcome vaccine hesitancy, benefits of vaccinating, and disadvantages of not getting vaccinated.

#### Concerns about the COVID-19 vaccines

##### Vaccine safety

Concerns about vaccine safety were mentioned in all focus group discussions. When participants were asked about COVID-19 vaccine saftey, one participant said: “Well, nothing is 100% safe. Not even the vaccines, I think, that’s why they don’t always tell you 100% that it’s safe; they tell you 95% or 99%”. In contrast, another participant responded that the vaccine’s safety had been used as a tool to control the population before vaccination: “How can I be sure that something is being sold as good or bad? It is simply to calm the population so the panic will go away (…) personally; I am not sure it’s safe”.

##### Vaccine effectiveness

Participants were unsure whether the COVID-19 vaccines were effective. A participant said that if the vaccines were effective, they would be worth fighting for: “If the vaccines were effective, I think that rather, we would fight for it. Yes, but as we already know, it is not (the case…)”. Another participant said that vaccinated and unvaccinated people carry the same risk of getting COVID-19. Therefore, it is essential to consider whether to get vaccinated: “You carry the same risk of getting sick as people who have not been vaccinated. So, there are things that one has to think about”. Another participant said, “the effectiveness, that’s what worries the most”. For others, vaccines are not helpful if they cannot prevent you from getting sick with the disease: “what is the use of the vaccine if it will not free us from COVID?”.

#### Feelings of fear

##### Fear of the vaccine effectiveness

Participants were afraid because they believed vaccines are ineffective. Even though you get vaccinated, you can still get sick with COVID-19: “It’s only fear and effectiveness”, another said, “that is the fear we have if the vaccine does not work”.

##### Fear of reinfection

Participants were frightened of getting infected/reinfected with COVID-19 after being vaccinated: “The truth is that nobody feels safe… and no one can assure me that by getting the vaccine, COVID will no longer affect me. I have relatives who have been vaccinated and had COVID afterward. So that’s why”. Another participant said they fear getting the disease with stronger symptoms after being vaccinated: “My father, his wife, and their son got vaccinated. And it affected them again even harder (meaning that they had COVID again and had stronger side effects). Moderator: Did they get COVID-19 after getting the vaccine? “Yes”.

##### Fear about the safety of the vaccine

We asked how people in the community felt about the COVID-19 vaccine. A participant said that people in their community do not feel safe because they fear the vaccine: “I know how most people feel about getting it. They are afraid of the vaccine. They do not feel safe because they fear getting the vaccine”. Others fear dying because of the vaccine: “With the injection (COVID-vaccine), people have even died, according to the radio, and that is the fear we have”.

##### Fear of the vaccine’s side-effects

We asked about the vaccine side effects. A participant shared her adult daughter’s experiences with the vaccine:

“Mother, she said, with the second dose, it was as if a truck had run over me. It gave her a fever. There wasn’t a bone in her body that didn’t hurt. She got really bad; she couldn’t go to work. For 2 days, she was in bed, and I was really scared. Because –we really don’t know what they’re giving us. That is the real concern”.

Participants question whether it is worth the risk of getting vaccinated: “People said why to get the vaccine if I am going to be sick. A person I know got vaccinated. He texted me in the afternoon, you know I have a high fever, like the flu. I got vaccinated in the morning, and he told me; I could not believe this. So, you think there will not be such strong reactions; when you hear that there are, it is best not to get the vaccine because what if I get it? What if something happens to me, right? I don’t know”.

#### Knowledge about the COVID-19 vaccines

##### Locations where to get a COVID-19 vaccine

Most participants knew where to get the vaccine: “Well, I have been told in CVS or Walgreens. You have to go to a Facebook page to make an appointment. Or any pharmacy”. Other participants said “clinics” and “Wal-Mart”.

##### Personal opinions vs. scientific facts

We asked about the ideal person to convey messages about the vaccine. A participant said doctors, but she said that some doctors provide confusing messages: “Because there is a doctor, who, although he is a doctor, presents information that is sometimes distorted. It confuses people. Some doctors shared their personal opinion; the information must be reliable. But giving your personal opinion is what confuses”.

Some participants don’t know how the vaccine works. Their knowledge can be based on a misconception: “They don’t specify what the vaccines are made of, but I imagine they must have the COVID viruses; I do not know how because those were the vaccines they gave us previously. The vaccine was for measles”.

##### Sources of information about COVID-19 vaccines

Most participants use social media platforms as their sources of information: “On the Internet”, “More than anything, the news”. Another participant said, “Google”, and another one, “Facebook”. Participants revealed that contradictory messages among the news, the head of CDC, and the government were barriers to understanding the importance of vaccination: “Fauci contradicts himself, and then you see him and say, Oh, wow! If he is the one, who knows… And he says one thing one day and another thing the next day, so that’s when you doubt”. “There is no agreement between what the news and the government report about the vaccines”.

#### Benefits of getting a COVID-19 vaccine

Not all participants agreed upon the benefit that you can get by vaccination. “You always catch the disease despite being vaccinated. There is no advantage (…) I can get COVID again”. Another participant said, “It will affect me (getting sick with COVID) if I take it; if I don’t, the same results. There is no guarantee that you will not get sick”. But for others, the vaccine can save lives. With the vaccine, you can gain personal benefits and/or societal benefits: “To save lives… one protects oneself, protects the family, and protects other people”. Another participant believed that vaccines would lessen the effect of the disease: “Well, in any case, if you get COVID, that does not affect you strongly”.

#### Disadvantages of not getting vaccinated

Only one participant talked about the disadvantage of not getting the vaccine would be the risk of dying: “That you can die from COVID”. Others expressed their concern about getting sick with the disease and having to go to a hospital in the US:

“In situations where you get seriously ill and need to go to the hospital or something. For example, what will we do if we do not have health insurance? My husband had some tests done in March, so we have to pay $12,000. Someone says if I’m fine, I’d rather take care of myself and protect myself”. Meaning that they will get the vaccine as soon as they can. Further, many participants believe that getting sick in this country is a luxury they cannot afford since healthcare is highly costly: “What happens is that it’s a luxury, getting sick here (in the US). We can’t afford that luxury”. Another participant said, “It’s expensive, and what will I do?”

#### Suggestions to overcome vaccine hesitancy

We asked about the ideal message, messenger, and medium to address COVID-19 vaccine concerns and misconceptions.

##### Messages

Participants desired that COVID-19 vaccines were the cure for the disease, meaning to increase their effectiveness: “That the vaccine was the cure, not just getting vaccinated for the sake of getting vaccinated, right? But if you know that you will get vaccinated and this is the cure, then you will get vaccinated”. They also expressed the need to ensure the safety of the vaccine ingredients to reduce the intense side effects, even death: “More safety regarding the vaccine ingredients, right? I fear the side effects; I have heard many things: it gives you fever (…) I already had COVID, and with a vaccine, it will leave me like this again”. Meaning that she will be sick again.

### Results of the follow-up survey

Most participants (80%) got at least one COVID-19 vaccine. Of those, over one-half received a two-dose vaccine (57%), while 43% completed their vaccination series (since they received a one-dose vaccine). Of those receiving a two-dose vaccine, only 50% have completed their vaccination series. When we asked whether they would be planning on getting the second dose, participants responded that they were not planning to get a second dose. Of those who responded that they did not get vaccinated (20%), reasons were because they were unsure and not planning on doing it for personal reasons.

## Discussion

The results of our mixed-method study with Hispanic adults living in rural regions of South Carolina (SC) revealed potential facilitators and barriers to COVID-19 vaccination. Participants shared their concerns about the vaccines’ effectiveness, safety, side effects, and even fear of death despite vaccination. Similar results were found by Knight et al. [17]. Their main reasons for hesitancy were concerns about side effects, the speed of vaccine development, and people being unsure about the vaccine’s effectiveness [17]. Our study also revealed that participant’s feared getting sick with the disease and reinfection, especially since they felt they could not afford healthcare or take time off work to be sick. These results were unique to our study. Regarding the main barriers to vaccination, some of our study participants responded that fear and vaccine effectiveness were the only obstacles to immunization. These results contradict the literature that suggested lower rollout of COVID-19 vaccines to minority communities [12, 17, 18].

For sources of information about the COVID-19 vaccines, our participants shared using social media platforms such as Facebook, TikTok, the Internet, Google, and the news. They also reported conflicting/contradictory messages from the media outlets, the head of the CDC, and the government as a barrier to understanding the importance of the vaccine and the measures needed. Lack of adequate information and misinformation about the vaccines from unreputable sources could be one of the main reasons for vaccine hesitancy among our participants. Kricorian and Turner [8], who had similar results, explained that the lack of trust and willingness to receive the COVID-19 vaccine might be due to inadequate information or the spread of misinformation about the vaccine among minority communities [8]. Similar results were found by Schilling et al. [9]. Their study showed that misinformation is still a problem for vaccine decisions [9].

Regarding the vaccine benefits, many participants did not perceive any benefits because they did not believe it was effective. This was partly due to the availability of a third or booster dose of the vaccine, yet people can still get sick with the disease. However, others believed it could save a life or lessen the effect of the disease. Schilling et al. [9] shared similar results regarding the benefits and risks of vaccines (e.g., efficacy, safety, return to normalcy, and protecting loved ones) [9].

Even though most of our study participants were unsure about the safety and effectiveness of the vaccine at the moment of the intervention, our online follow-up survey results revealed that 80% got at least one vaccine. Of those, 57% received a two-dose vaccine, and 50% completed their vaccination series. Limited research has been found in the literature that proposed culturally sensitive interventions to address COVID-19 vaccine hesitancy.

Based on these results, the educational component we implemented with Hispanics living in rural areas of SC seemed to be an effective way to relay information about the COVID-19 vaccine and dispel myths. While an increase in vaccination rates would be expected over a 6–8 months period as an effect of time, the magnitude of change in the vaccination rate suggests that our intervention may have been effective for delivering vaccine education and directly addressing myths about vaccination as a strategy to increase vaccination within a Hispanic population.

Limited research has been found in the literature that proposed culturally sensitive interventions to address COVID-19 vaccine hesitancy. Chen et al. [19] are conducting a randomized control trial in the US and China to compare the effectiveness of four different approaches: a storytelling-instructional-humor, a storytelling-analogy, a storytelling emotion-focused approach, and no video. The authors will measure vaccine hesitancy, focusing on COVID-19 vaccine hesitancy as the main outcome and behavioral intent to seek vaccination and hope as secondary outcomes. Since these videos are wordless and culturally inclusive, they would be accepted by all cultural groups [19]. However, there are no published results since this is an ongoing study.

Another ongoing research project is also a clinical trial conducted with Hispanics and African American communities in Southern California by Servin et al. [20]. It is a multidimensional community intervention and the main outcomes measured are changes in COVID-19 vaccine acceptance and changes in COVID-19 vaccine hesitancy. No results have been published yet [20].

### Study limitations

The current study has several limitations. A homogenous convenience sampling technique was used for participant recruitment. This type of technique can lead to a selection bias in which some members of a population are more likely and willing to participate than others which limits its generalizability [21]. Even though we excluded the response of six vaccinated individuals from data analyses, they may have influenced others in their opinions during focus group discussions. We only explained how the mRNA vaccine works (Pfizer) since it had full FDA approval at the time of the intervention. It is possible that people may have considered other vaccines manufactured by other companies. Therefore, we did not address questions specific to other vaccines. The recommended number of focus groups needed to capture 90% of the themes and sub-teams used was appropriate [22]. Even though the recommended size for a focus group discussion is between 5–10 participants [23, 24], the number of participants per focus group was smaller than we expected. This was a complex mixed methods evaluation study to measure the impact before and after program participation. In this type of study, there may not have been enough time to observe modifications in participants’ behaviors [25],– however, in our study, education seemed to influence vaccination status at 6 months since most participants (80%, *n* = 8) had received at least one dose.

## Conclusion

The study revealed that hesitancy among Hispanics living in rural South Carolina originated from misinformation and unreliable sources. It was also rooted in participants’ perceptions about the vaccine’s safety and effectiveness, including perceptions that vaccine development was rushed and questionable ingredients were used in making the vaccine that could produce substantial side effects and can cause death. Participants feared reinfection or going to the hospital since this was a luxury they could not afford. The education component seemed to influence vaccination since 50% of our participants completed their vaccination series, 20% self-reported receiving at least one dose, and 10% (*n* = 1) self-reported to be vaccinated but did not report the type of vaccine received.

## Practical recommendations

Public health officials should ensure information is provided in Spanish during the vaccination process, including advertising, COVID-19 vaccine health communication, and outreach messaging. Participants shared different mediums they would like to receive information about vaccines, such as videos on social media, Facebook, YouTube, and TikTok. Also, round table discussions or school meetings. Flyers or written information are not preferred unless the information is in a text message form so they can read it from their cellphones. To dispel mistrust, participants perceived the ideal messengers to provide information would be doctors, who are studying the disease, and researchers because they are neutral. They also recommend presenting scientific facts and reliable information, not personal opinions. The message should address how the vaccines were developed and worked. Program implementation and mass communication campaigns to address vaccine hesitancy should focus on and provide an interpretation of vaccines’ effectiveness, safety, and how the vaccine works. Another recommendation was to increase vaccine effectiveness and safety. Further research should focus on ways to increase confidence in vaccine effectiveness and safety.

### Supplementary Information

**Additional file 1.** Focus group protocol.

## Data Availability

The datasets used and/or analyzed during the current study are available from the corresponding author upon reasonable request.

## Abbreviations

COVID-19: SARS-CoV-2, 2019 virus that causes COVID-19
CDC: Center for Disease Control and Prevention
