# Supplementary material for: Understanding COVID-19 vaccine hesitancy in the Hispanic adult population of South Carolina: a complex mixed-method design evaluation study
Source: BMC Public Health. 2023 Nov 28;23:2359. doi: 10.1186/s12889-023-16771-9 (PMC10685550; doi:10.1186/s12889-023-16771-9)
Supplement: Supplementary file 1 — Additional file 1. Focus group protocol. [file 12889_2023_16771_MOESM1_ESM.docx]

**Focus group protocol**

**Introduction (10 minutes) Facilitator [*READ*]:**

Thank you for taking the time to meet with us today. My name is _______, and I will be asking you a few questions about COVID-19 vaccination. This is ___________ who will be taking notes on what we talk about so that we can make sure not to miss any of your thoughts.

We are talking to you today because we’d like to understand how you and your community feels about the COVID-19 vaccines, what information you have heard, and what information you would like to have in the future. When we say “community,” we mean your friends and family members and others you may know in your everyday lives. We are going to ask you some questions and then have a discussion where we hope to hear from everybody. There are no right or wrong answers. The things we learn from you will be important to making sure you and the people in your community get the information they need about the COVID-19 vaccine. This discussion will take about one hour and 15 minutes.

This discussion is voluntary, meaning that you are answering these questions because you want to, not because you have to. If you prefer not to answer a question, it’s totally fine. You can also leave the discussion at any time. This will not impact you in any way.

We may share the aggregate data or the general information that we learn, publicly so that others may learn from this discussion. This may include publishing the results externally. If you agree to participate in this discussion, we will be using your demographic information in our reports, including your gender, age, race, and ethnicity. This will help us to understand differences in responses. No names or personally identifiable information will be shared in reports or publications.

We are asking everyone here to please keep what others share private so that everyone can talk openly and honestly. Also, we want you to ask questions or let us know if you don’t understand something.

We are recording this discussion so we can take notes and not miss anything that was said. The recording will also be transcribed by a translation company after the session. The recording will not be shared with anyone outside of the translation company or our team, and it will be deleted after the translation company has transcribed it. However, we will be keeping the transcript of the recording and the notes taken by the notetaker. Notes and transcripts will also be translated into English by our translators. Your personally identifiable information will not be included anywhere in the transcript or notes. Notes and transcripts will be shared with the translators and our team.

Thank you so much for being here and being willing to do this! Your thoughts and ideas are really helpful in fighting the pandemic.

Before we get started, do you feel comfortable participating in this discussion and answering my questions? Do you have any questions before we start?

I want to highlight three key points again.

- Your name and personally identifiable information will not be shared in any reports.
- Your participation in this interview is voluntary.
- You can stop answering questions or leave at any time. This won’t impact you in any way.

Do you understand, and do you agree to continue?
Informed consent was obtained from participants: □ Yes □ No

**Focus Group Discussion Questions (1 hour)**

I would first like to take two minutes to meet each other. Please share your name, your favorite hobby and tell one or two ways that Covid-19 affected your everyday lives. [Prompt participants to share.]

We will now start the recording (ensure Note Taker starts recording).

I want to ask you to think about your community – friends and family members and others who you interact with in your daily lives.

1. Overall, how do you think your community feel about the COVID-19 vaccine? (15 minutes)
   1. Probe: Do you think that most people in your community will get the vaccine? Why or why not?
   2. Probe: What do you or your community see as the benefits of getting vaccinated against COVID-19?
   3. Probe: What do you or your community see as the downsides of getting vaccinated against COVID-19?
   4. Probe: How do people in your community feel about the safety of the Covid-19 vaccines?
   5. Probe: How do people in your community feel about the effectiveness of the Covid-19 vaccines?
2. What information have you and people in your community heard about the COVID-19 vaccine? (15 minutes)
   1. Probe: What kind of stories have you heard about the COVID vaccine?
   2. Probe: What does it contain and how does it work?
   3. Probe: What is your understanding of the side effects?
   4. Probe: Where can you get the vaccine? How hard is it to get the vaccine?
   5. Probe: What happens once you receive the vaccine? Do you still have to wear a mask or keep distance?
3. What would prevent you and members of your community from getting the vaccine? (10 minutes)
   1. Probe: What concerns do you/your community have about the vaccine?
   2. Probe: What barriers could keep you from getting the vaccine? (e.g., access, childcare, transportation, etc.)
4. For people in your community who might be hesitant about getting the vaccine, what would encourage them to get it? (10 minutes)

a. Probe: What would make [your community] feel safe about getting a COVID-19 vaccine?

b. Probe: What information do people in your community wish they had about the vaccine?

5. What would you recommend as the best way to communicate information about COVID-19 vaccines to your community? (10 minutes)

1. Probe: Who would be the best person to share information about the vaccine or help teach people about a COVID-19 vaccine? Healthcare providers, family, friends, religious leaders?
2. Probe: What are the best ways to reach people in your community? (e.g., face-to-face, WhatsApp, Facebook, email, mail, phone/text, YouTube?)
3. Probe: Where do you and people in your community get information about COVID-19 vaccines?
4. Probe: Does your community prefer information to be written or spoken?
5. Probe: The Clemson Extension Service works in communities across the state. How could Clemson Extension help people in your community to learn more about the Covid-19 vaccine and/or get vaccinated?

**Closing (5 minutes)**

This concludes the questions. Thank you again for participating in this discussion today. We know that your time and ideas are valuable, and we would like to give each of you a ____ gift card to thank you. You will receive this gift card via email or text message, and I will follow up with you by next week to confirm that I have your correct information. If you have any questions following this discussion, you can contact me at ____________. Thank you.
